# Efficacy of Tumor Necrosis Factor-α Inhibitor Adalimumab in Chronic Recurrent Vogt–Koyanagi–Harada Disease

**DOI:** 10.3390/ph18121848

**Published:** 2025-12-03

**Authors:** Junghoo Lee, Yoo-Ri Chung, Hae Rang Kim, Ji Hun Song

**Affiliations:** Department of Ophthalmology, Ajou University School of Medicine, Suwon 16499, Republic of Korea; junghoo9999@naver.com (J.L.); khr1412@hanmail.net (H.R.K.)

**Keywords:** adalimumab, corticosteroid, immunosuppressive agent, tumor necrosis factor-α inhibitor, uveitis, Vogt–Koyanagi–Harada disease

## Abstract

**Background/Objectives:** Vogt–Koyanagi–Harada (VKH) disease is a bilateral granulomatous panuveitis that can progress to a chronic, relapsing phase. Patients refractory or intolerant to systemic corticosteroids and conventional immunomodulatory therapy pose a major therapeutic challenge, as persistent inflammation can lead to cumulative ocular damage and permanent vision loss. This study assessed the efficacy of tumor necrosis factor-α (TNF-α) inhibitor adalimumab in chronic recurrent VKH disease. **Methods:** We retrospectively reviewed 16 eyes from 8 patients with chronic recurrent VKH disease who had persistent inflammation despite treatment with corticosteroids and conventional immunomodulatory therapy, and subsequently received adalimumab. Primary outcomes were changes in subfoveal choroidal thickness (SFCT) and systemic corticosteroid dose reduction. Secondary outcomes included visual acuity, inflammatory parameters (anterior chamber cell, flare, and vitreous haze), and central macular thickness (CMT). All outcomes were compared between baseline and 6 months after adalimumab initiation using the Wilcoxon signed-rank test. **Results:** Mean patient age was 47.6 years and mean follow-up was 31.8 months. SFCT decreased from 326.7 ± 129.1 µm to 231.6 ± 72.9 µm at 6 months (*p* < 0.001). Systemic steroid dose decreased from 14.7 ± 14.0 mg to 4.1 ± 3.8 mg (*p* = 0.027). Mean annualized relapse rate decreased from 3.61 to 0.08 episodes/year (*p* = 0.012). Anterior chamber cell grade decreased from 0.81 ± 0.66 to 0.09 ± 0.20 (*p* < 0.001). Visual acuity, flare, vitreous haze, and CMT showed no significant change. No serious adverse events occurred. **Conclusions**: TNF-α inhibition with adalimumab appears effective as steroid-sparing therapy for controlling recurrent inflammation and reducing steroid dependence in patients with chronic recurrent VKH disease refractory to conventional treatment.

## 1. Introduction

Vogt–Koyanagi–Harada (VKH) disease is a multisystem autoimmune condition characterized by bilateral granulomatous panuveitis often accompanied by neurologic, auditory, and cutaneous manifestations. Acute VKH disease can initially be controlled with prompt high-dose corticosteroid therapy; however, many patients progress to a chronic recurrent stage in which persistent intraocular inflammation leads to cumulative structural damage and vision loss. This chronic, relapsing phase of VKH disease poses a significant therapeutic challenge [1,2].

Conventional management relies on the use of prolonged systemic corticosteroids, often combined with immunomodulatory agents (e.g., methotrexate, mycophenolate mofetil, or cyclosporine). While these treatments can suppress inflammation, extended use is associated with serious adverse effects. High-dose corticosteroids may cause significant metabolic, musculoskeletal, and ocular toxicities, while steroid-sparing agents carry risks of infection, organ damage, and malignancy [3]. Moreover, some patients remain refractory (persistent inflammation despite therapy) or intolerant (unable to continue therapy due to side effects) to these conventional treatments. These limitations highlight the need for safer and more targeted immunomodulatory strategies in chronic VKH.

From an immunological perspective, VKH disease is mediated by an autoimmune T-cell response targeting melanocyte-associated antigens. While T helper 1 (Th1) cells primarily drive the acute phase, T helper 17 (Th17) cells play a major role in chronic and recurrent inflammation [4,5]. Among the inflammatory mediators involved, tumor necrosis factor-α (TNF-α) plays a pivotal role in VKH pathogenesis by promoting leukocyte recruitment and tissue infiltration [6,7,8]. Studies have demonstrated markedly elevated TNF-α levels in the aqueous humor of VKH patients—approximately 10-fold higher than in healthy controls—particularly in those with recurrent disease patterns [8,9]. This immunological framework provides a strong rationale for targeting TNF-α in managing this chronic, refractory condition.

Adalimumab (ADA), a humanized recombinant monoclonal antibody, binds specifically to TNF-α, blocking its interaction with TNF receptor 1 (TNFR1) and TNF receptor 2 (TNFR2), and neutralizing its proinflammatory effects. By modulating the TNF-α–mediated inflammatory cascade, ADA has demonstrated clinical efficacy as a steroid-sparing agent in noninfectious uveitis [10]. The clinical utility of ADA in reducing uveitic flares and corticosteroid dependence has been demonstrated in pivotal trials, including VISUAL-1 and VISUAL-2, with VISUAL-3 further supporting its long-term safety and efficacy, including its use in patients with VKH disease [3,6,11].

This study was conducted to evaluate the efficacy and safety of ADA in patients with chronic recurrent VKH disease, with particular focus on those with severe steroid intolerance or refractoriness to conventional therapy.

## 2. Results

### 2.1. Baseline Characteristics

A total of 8 patients (16 eyes) with chronic recurrent VKH disease refractory to conventional therapies were included in this retrospective study. Baseline demographic and key clinical characteristics, including prior relapse rates, are summarized in Table 1. The mean age at ADA initiation was 47.6 ± 9.5 years (range: 30–59), with three males (37.5%) and five females (62.5%).

At baseline, the mean daily prednisone-equivalent dose was 14.7 ± 14.0 mg. Six patients (75%) were undergoing systemic corticosteroid therapy at ADA initiation, while the remaining two (25%) had discontinued steroids due to severe intolerance. All patients were classified as refractory to conventional therapy due to either steroid intolerance or persistent inflammation despite prior treatments. Previous immunomodulatory therapies (IMT) included mycophenolate mofetil (*n* = 6), cyclosporine A (*n* = 5), and methotrexate (*n* = 1), often in combination regimens. Before starting ADA, all patients had experienced multiple uveitic relapses, with a mean annualized relapse rate of 3.61 ± 3.55 episodes/year (Table 1).

Detailed individual data, including prior treatment regimens, documented adverse effects, and pre-ADA relapse histories, are presented in Table 2. Notably, this cohort includes patients with severe steroid intolerance, such as Patient 5 (who experienced generalized edema and steroid-induced psychosis) and Patient 7 (Cushing syndrome). It also includes patients with exceptionally high pre-treatment disease activity, such as Patient 4 (annualized relapse rate [ARR] of 9.00) and Patient 5 (ARR 8.00).

### 2.2. Primary and Secondary Outcome Analysis

**Figure 1:**
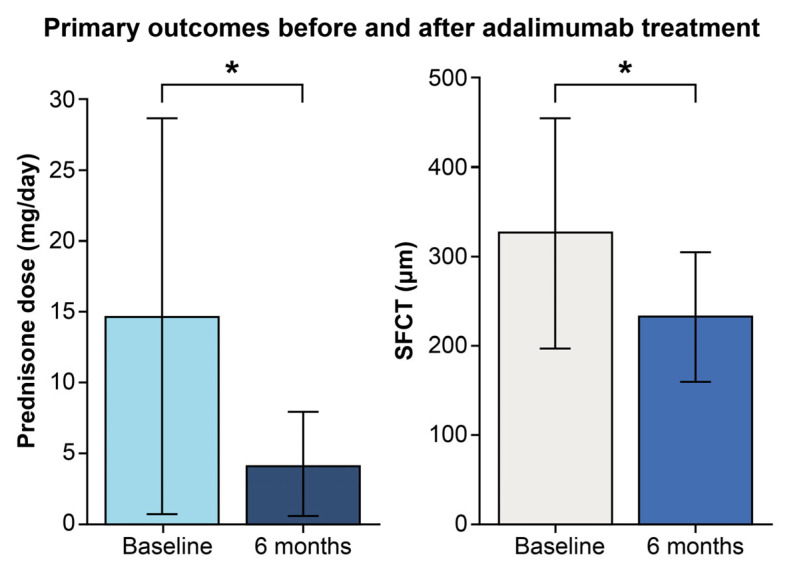
Steroid-sparing effect and choroidal thickness reduction with adalimumab in chronic Vogt–Koyanagi–Harada (VKH) disease. Primary outcomes before and after adalimumab therapy in patients with chronic VKH disease. Left: Mean daily prednisone dose significantly decreased following treatment (*p* = 0.027, paired *t*-test). Right: Mean subfoveal choroidal thickness significantly decreased at 6 months, reflecting resolution of choroidal inflammation (*p* < 0.001, Wilcoxon signed-rank test). Error bars represent standard deviations. Asterisks (*) indicate statistically significant differences (*p* < 0.05).

The primary outcome was the change in the daily prednisone-equivalent dose from baseline to 6 months (N = 8 patients). The Shapiro–Wilk test showed no significant deviation from normality (W = 0.907, *p* = 0.332), allowing for paired *t*-test analysis. The mean daily dose significantly decreased from 14.7 ± 14.0 mg at baseline to 4.1 ± 3.8 mg at 6 months, with a mean reduction of 10.6 mg [95% CI: 1.64 to 19.61 mg; t (7) = 2.795, *p* = 0.027] (Table 3 and Figure 1). A Wilcoxon signed-rank test was also performed due to the small sample size and confirmed the significant reduction (Z = −2.201, *p* = 0.028). By 6 months, three patients (37.5%) had discontinued systemic corticosteroids, and four others (50%) maintained low-dose regimens (≤5 mg/day).

Another key outcome, the change in subfoveal choroidal thickness (SFCT) over 6 months (N = 16 eyes), also showed significant improvement. As SFCT differences were not normally distributed (Shapiro–Wilk W = 0.862, *p* = 0.021), the Wilcoxon signed-rank test was used. The mean SFCT decreased from 326.7 ± 129.1 µm at baseline to 231.6 ± 72.9 µm at six months (Z = −3.362, *p* < 0.001) (Table 3 and Figure 1).

**Figure 2:**
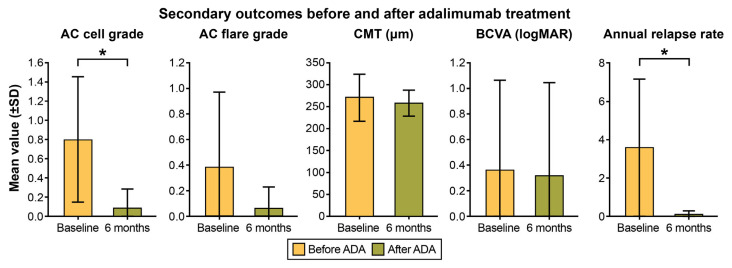
Secondary outcomes before and after adalimumab treatment in chronic recurrent Vogt–Koyanagi–Harada (VKH) disease. Changes in the anterior chamber (AC) cell grade, flare grade, central macular thickness (CMT), best-corrected visual acuity (BCVA), and annualized relapse rate (ARR) are shown. Significant improvements were observed in AC cell grade (*p* < 0.001) and ARR (*p* = 0.012), while other parameters did not reach statistical significance. Error bars represent standard deviations. Asterisks (*) indicate statistically significant differences (*p* < 0.05).

Significant improvements were also observed in key secondary outcomes. A significant improvement was observed in anterior segment inflammation control, with the mean anterior chamber (AC) cell grade decreasing from 0.81 ± 0.66 to 0.09 ± 0.20 (*p* < 0.001, Wilcoxon signed-rank test) (Table 3 and Figure 2). Furthermore, ADA therapy significantly reduced disease activity. The mean annualized relapse rate dropped markedly from 3.61 ± 3.55 episodes/year before ADA to 0.08 ± 0.16 episodes/year after treatment (Z = −2.521, *p* = 0.012) (Figure 2).

Changes in other secondary outcomes did not reach statistical significance. Mean best-corrected visual acuity (BCVA) changed slightly from 0.36 ± 0.70 logarithm of the minimum angle of resolution (logMAR) at baseline to 0.32 ± 0.72 at 6 months (*p* = 0.084). AC flare exhibited a numerical decrease from 0.38 ± 0.59 to 0.06 ± 0.17 (*p* = 0.059), and central macular thickness (CMT) decreased slightly from 270.8 ± 54.2 µm to 257.5 ± 29.9 µm (*p* = 0.325). An exploratory Spearman rank correlation between SFCT reduction and BCVA change at 6 months demonstrated no significant association (rho = −0.156, *p* = 0.564) (Table 3 and Figure 2).

### 2.3. Representative Cases

**Figure 3:**
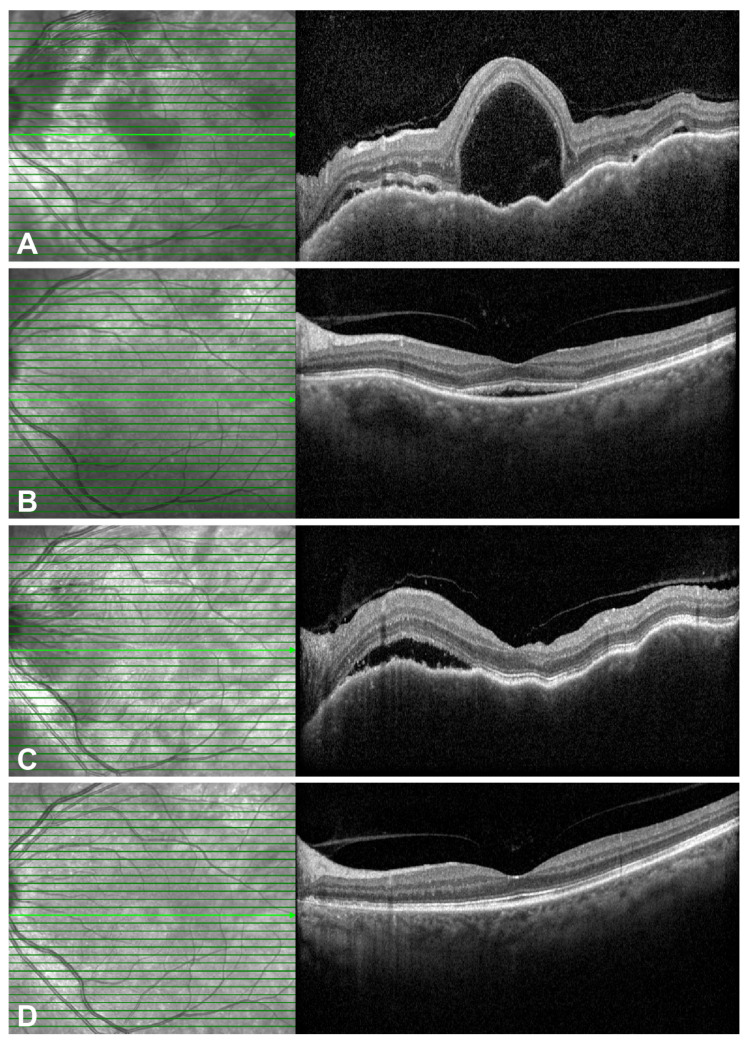
Serial optical coherence tomography (OCT) images from a patient with chronic recurrent Vogt–Koyanagi–Harada (VKH) disease who developed severe systemic side effects to conventional therapy. (**A**) At initial presentation, the OCT shows significant subretinal fluid (SRF) and increased choroidal thickness with an undulating retinal pigment epithelium layer. Treatment with high-dose systemic corticosteroids and cyclosporine was initiated. (**B**) One month later, despite a significant reduction in the SRF, conventional therapy was discontinued due to severe adverse effects, including generalized edema and steroid-related psychosis. (**C**) Following treatment cessation, a rapid recurrence of SRF with increased choroidal thickness was observed 1 month later, at which point adalimumab monotherapy was initiated. (**D**) After 1 month of adalimumab treatment, the SRF completely resolved. The patient remained stable without relapse for the subsequent 18 months, demonstrating effective long-term disease control with adalimumab alone. The green arrow indicates the level of the optical coherence tomography scan.

**Figure 4:**
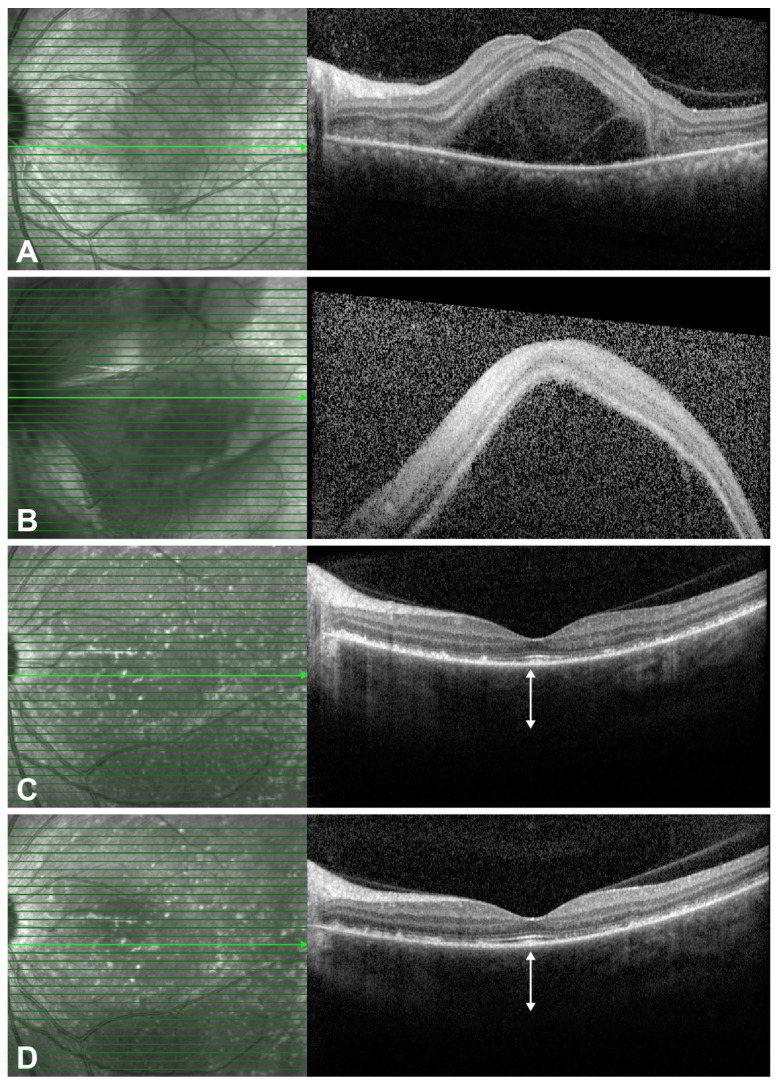
Serial optical coherence tomography (OCT) images from a patient with highly refractory chronic recurrent Vogt–Koyanagi–Harada (VKH) disease. (**A**) The initial OCT shows serous retinal detachment with subretinal septation, unresponsive to systemic corticosteroids and mycophenolate mofetil, leading to a posterior subtenon steroid injection (PSTI). (**B**) One week post-PSTI, the subretinal detachment worsened significantly, necessitating intravitreal dexamethasone implant treatment. (**C**) Three months after the implant, despite improvement in the subretinal fluid, other signs of active inflammation (AC cells, disc hyperemia) emerged, and the patient developed significant corticosteroid-related side effects. Adalimumab therapy was subsequently initiated. (**D**) One month after starting adalimumab, a marked anatomical improvement with resolution of all inflammatory signs and decreased choroidal thickness (double-headed arrow) was observed, correlating with an improvement in visual acuity to 20/20. The green arrow indicates the level of the optical coherence tomography scan.

The clinical utility of ADA is further illustrated by two representative cases. In the first case (patient 5, detailed in Table 2), a patient intolerant to conventional therapy due to adverse effects (e.g., generalized edema, steroid-induced psychosis) achieved complete anatomical resolution and an 18-month remission on ADA monotherapy, highlighting its steroid-sparing potential (Figure 3). The second case (patient 6, detailed in Table 2) demonstrated ADA’s efficacy as a rescue agent in a highly refractory patient, achieving full resolution of inflammation after failure of multiple prior therapies (Figure 4).

## 3. Discussion

### 3.1. Clinical Efficacy and Steroid-Sparing Effect in the Current Study

Our findings demonstrate that TNF-α inhibition with ADA, often administered alongside conventional IMT, effectively controls inflammation and reduces corticosteroid dependence in patients with chronic recurrent VKH disease refractory to conventional treatments. In this cohort, ADA treatment led to a significant reduction in intraocular inflammation, as shown by decreased AC cell grades and SFCT, while visual acuity generally remained stable.

Importantly, the mean daily corticosteroid dose was reduced from 14.7 mg to 4.1 mg (*p* = 0.027), with 37.5% of patients able to discontinue corticosteroids entirely and another 50% maintained on a low dose (≤5 mg/day). These findings highlight the steroid-sparing potential of ADA, which may help mitigate long-term corticosteroid-related complications.

Furthermore, ADA therapy significantly reduced disease activity, with the annualized uveitic relapse rate declining from 3.61 to 0.08 episodes/year. This suggests that sustained TNF-α inhibition may suppress recurrent inflammatory activity and promote long-term disease quiescence. Collectively, these results support ADA as a viable therapeutic strategy to improve outcomes and reduce corticosteroid exposure in this vision-threatening condition.

### 3.2. Immunopathological Rationale for Adalimumab Therapy in VKH Disease

Among the cytokines implicated, TNF-α plays a pivotal role by promoting leukocyte recruitment, endothelial activation, and tissue infiltration [6,7]. Cytokine profiling of aqueous humor has shown markedly elevated TNF-α levels in patients with VKH disease than in healthy controls (139.7 ± 66.6 pg/mL vs. 14.2 ± 3.6 pg/mL; *p* < 0.001), underscoring its key contribution to disease pathogenesis [8]. Elevated TNF-α levels are also found in recurrent-pattern uveitis, suggesting that relapsing or refractory VKH disease may be associated with persistent TNF-α–driven inflammation [4].

At the molecular level, TNF-α binds to TNFR1 and TNFR2, activating intracellular pathways that induce transcription of additional proinflammatory mediators such as interleukin (IL)-1β, IL-6, and various chemokines [7,12]. This inflammatory cascade is further exacerbated by impaired T-cell (Treg) function, which fails to suppress the aberrant Th1/Th2 response. Notably, TNF-α–induced IL-6 production is critical, as IL-6 promotes Th17 cell differentiation and pathogenicity, contributing to disease chronicity [13].

This immunological framework provides a strong rationale for targeted anti-TNF-α therapy with ADA. Its mechanism is twofold: ADA directly neutralizes TNF-α, preventing downstream effects such as leukocyte migration and endothelial activation, and indirectly modulates the Th17 axis by suppressing IL-6 production. By disrupting the cytokine environment necessary for Th17 differentiation and function, ADA offers targeted control of both initiating and amplifying inflammatory processes [5,14,15].

### 3.3. Comparison with Previous Literature and the Role of Combination Therapy

The efficacy of ADA in managing noninfectious uveitis (NIU) has been well-established through landmark clinical trials such as the VISUAL I, II, and III studies [3,6,11]. These trials demonstrated that ADA significantly reduces the risk of treatment failure, controls uveitic flares, and enables corticosteroid sparing action in patients with active or inactive NIU, including intermediate uveitis, posterior uveitis, and panuveitis. Building on this foundation, several studies have specifically investigated the role of ADA in VKH disease.

Consistent with its broader efficacy in NIU, our findings and those from previous studies support the utility of ADA in managing refractory VKH disease. Yang et al. reported substantial reductions in the AC cell grades (median 2+ to 0.5+) and vitritis (median 1+ to 0), along with a decrease in mean prednisone dose from 21.91 mg/day to 2.73 mg/day in nine Chinese patients with refractory VKH disease [5]. Similarly, a multicenter study by Takeuchi et al. involving 50 patients with chronic recurrent VKH disease and sunset glow fundus demonstrated that ADA significantly improved the logMAR visual acuity, flare counts, SFCT, and indocyanine green angiography scores at 6 months, while reducing the mean corticosteroid dose from 16.5 mg/day to 7.05 mg/day [9]. Couto et al. also showed resolution of inflammation in 13 out of 14 patients, with the median corticosteroid dose decreasing from 20 mg to 4 mg following 6 months of ADA treatment [16]. Collectively, these findings underscore ADA’s effectiveness in controlling inflammation and enabling corticosteroid tapering in challenging VKH cases.

Table 4 summarizes the clinical outcomes from major recent studies of adalimumab therapy in VKH disease. This comparison reveals the therapeutic advantage of combining adalimumab with conventional IMT, particularly in chronic recurrent cases.

Multiple studies consistently report successful inflammation control when ADA is combined with IMT [5,9,16,17]. Most recently, Feng et al. corroborated the benefit of combining ADA with IMT, showing reduced recurrence rates in chronic recurrent VKH [18].

In contrast, ADA monotherapy in chronic recurrent VKH shows substantially higher relapse rates. Guo et al. found that while ADA reduced corticosteroid dependence in initial-onset VKH, all six patients in their recurrent subgroup relapsed [19]. Hiyama et al. also reported relapses in 78.6% (11/14) of monotherapy patients, whereas adding methotrexate reduced the relapse rate to 27.3% and extended the relapse-free interval [20]. These findings, consistent with our 87.5% remission rate in patients maintaining IMT with ADA, suggest that proactive continuation of concomitant IMT—rather than reactive addition after failure—provides superior control in chronic recurrent VKH.

This comparative evidence suggests a stage-dependent treatment approach. While initial-onset VKH may respond to ADA monotherapy, chronic recurrent VKH requires more potent and sustained anti-inflammatory control and benefits most from combination therapy (ADA plus IMT) [21]. The divergence in reported ADA efficacy across studies likely reflects these differences in patient selection and disease stage rather than drug efficacy itself.

**Table 4:** Comparison of Clinical Outcomes of Adalimumab (ADA) therapy in Key Vogt–Koyanagi–Harada (VKH) disease cohorts.

Study (Year)	*N* (Patients)	Steroid Dose Change (mg/day)		Key Outcomes	Concomitant IMT Strategy
		Baseline	Last Visit		
This Study	8	14.7	4.1	ARR, 3.61 → 0.08 (*p* = 0.012)	7/8 (87.5%)
Feng et al. (2024) [18]	28 (refractory subgroup) ^†^	53.33	2.43	Number of relapses, 1.43 → 0.36 (*p* = 0.009)	ADA added to ongoing conventional IMT in most patients
Guo et al. (2025) [19]	6 (refractory subgroup) ^†^	16.3	2.5	All 6/6 (100%) relapsed	5 with ADA + GC + IMT, 1 with ADA + IMT
Hiyama et al. (2021) [20]	14	N/A	N/A	ADA monotherapy: 11/4 (78.6%) relapsed	11/14 required MTX after relapse (ADA + low-dose MTX: 27.3%)
Takeuchi et al. (2022) [9]	50	16.5	7.05	Drug retention rate (94% remained on ADA)	22/50 (44%)
Yang et al. (2021) [5]	9	21.9	2.7	Median 1 relapsed (IQR 0–2)	9/9
Couto et al. (2018) [16]	14	20 (median)	4 (median)	Inflammation resolved: 92.8%	IMT use decreased (11 → 4 patients)

ADA, adalimumab; ARR, annualized relapse rate; GC, glucocorticoid; IMT, immunomodulatory therapy; IQR, interquartile range; MTX, methotrexate; N/A, not available; ^†^ Data for chronic–recurrent VKH subgroup are presented when available; these studies included both initial-onset and chronic cases.

### 3.4. Challenges and Study Limitations

Despite ADA’s efficacy in reducing inflammation, complete remission is not guaranteed in all cases, particularly those with chronic recurrent disease. Guo et al. reported that while ADA effectively reduced corticosteroid dependence in initial-onset VKH disease, it was less effective in preventing relapses in chronic or recurrent cases [19]. This underscores the challenge of managing long-standing disease and the influence of individual patient factors such as disease duration, prior treatments, and irreversible structural damage on outcomes. In our cohort, significant improvements were observed in the inflammatory and anatomical parameters, including the AC cell grade and SFCT. However, BCVA improved only modestly, from a mean of 0.36 to 0.32 logMAR (*p* = 0.084), failing to reach statistical significance. This limited visual recovery may be attributed to chronicity and irreversible structural changes such as photoreceptor or RPE damage in this refractory patient cohort. Additionally, the 6-month follow-up period may have been insufficient to capture delayed functional gains. The absence of a significant correlation between the changes in SFCT and BCVA (rho = −0.156, *p* = 0.564) further suggests that other factors beyond acute choroidal thickening influence the long-term visual outcomes.

Other secondary outcomes, including AC flare (*p* = 0.059) and CMT (*p* = 0.325), demonstrated numerical improvements that did not reach significance, possibly due to low baseline values and the small sample size. Moreover, the retrospective design and limited cohort size (8 patients, 16 eyes) precluded any a priori power calculation for the primary endpoint, and the study was not formally powered to detect modest treatment effects. Accordingly, the present results should be interpreted with caution and regarded as hypothesis-generating, pending confirmation in larger, prospective cohorts.

Despite these limitations, this study has several strengths. The novelty of this report lies in its specific focus on a small (*N* = 8) but highly characterized cohort with an extended follow-up (mean ≈ 39 months). Importantly, this cohort includes a clinically challenging subset often excluded from prior trials: patients who demonstrated severe steroid intolerance (e.g., psychosis, Cushing syndrome) to conventional therapies. Our standardized outcome assessments and calculation of annualized relapse rates allowed us to assess ADA’s role as a durable, steroid-sparing rescue therapy in this uniquely difficult-to-treat population. Our findings reinforce the potential benefit of concomitant IMT alongside ADA in chronic, treatment-refractory VKH disease.

### 3.5. Identifying the Appropriate Patient Profile for Adalimumab Therapy

A key clinical consideration arising from this study is the identification of the appropriate patient profile for ADA therapy in chronic recurrent VKH disease. Based on our findings, ideal candidates include patients who exhibit a refractory disease course with persistent inflammation despite treatment with high-dose corticosteroids and conventional immunomodulators, or those who experience recurrent inflammatory episodes during maintenance IMT. Furthermore, both our data and prior studies underscore the critical role of ADA for patients who become intolerant to conventional therapies due to significant side effects, such as Cushing syndrome or steroid-induced psychosis, warranting a change in the treatment strategy [3,17,22]. Expanding on our results, the literature suggests that patients presenting with a “sunset glow fundus”—indicative of chronic, widespread inflammation—also represent a patient profile that would derive particular benefit from the potent and sustained immunomodulation offered by ADA [9].

Based on our analysis and the comparative literature, two treatment paradigms can be considered: (1) for initial-onset VKH, ADA monotherapy or other conventional IMT may be sufficient; and (2) for chronic recurrent VKH, combination therapy (adalimumab with concomitant IMT) appears essential to control the inflammation and minimize relapse risk. Our data, showing an 87.5% remission rate with combination therapy versus high relapse rates reported with monotherapy in chronic disease, strongly support this stratified approach. Future prospective studies should directly compare these strategies to establish evidence-based treatment algorithms for different VKH disease stages.

## 4. Materials and Methods

### 4.1. Study Design and Ethical Approval

This retrospective review was approved by the Institutional Review Board of Ajou University Hospital (Suwon, Republic of Korea, approval no. AJOUIRB-DB-2025-214) and adhered to the tenets of the Declaration of Helsinki. The requirement for informed consent was waived due to the retrospective nature of the study.

### 4.2. Patient Selection

Patients were included if they met the revised diagnostic criteria for VKH disease, characterized by bilateral granulomatous uveitis with corroborative fluorescein and indocyanine green angiography findings, supported by spectral-domain optical coherence tomography (SD-OCT) [23]. The study focused on patients in the chronic recurrent stage, defined as those who initially achieved disease control with high-dose corticosteroids and conventional immunomodulatory agents but experienced one or more intraocular inflammation relapses during follow-up.

Patients unable to tolerate corticosteroids due to systemic side effects, or those with persistent or recurrent inflammation despite ≥3 months of treatment with systemic corticosteroids and at least one conventional immunomodulatory agent, were classified as having refractory VKH disease and considered for ADA therapy.

Exclusion criteria included active systemic infections (e.g., tuberculosis or viral hepatitis), a history of malignancy, other systemic autoimmune diseases, or significant ocular media opacities (e.g., dense cataract) that interfered with inflammation grading or imaging. Demographic and clinical data—including age, sex, disease duration, prior treatments, and ocular characteristics—were collected at baseline.

### 4.3. Treatment Protocol

All patients received subcutaneous ADA following the standard regimen for noninfectious uveitis: an 80 mg loading dose, followed by 40 mg after 1 week, and then 40 mg every other week as maintenance. At ADA initiation, 6 of 8 patients were undergoing oral corticosteroids (prednisone) therapy, while 2 had discontinued steroids due to intolerance.

Corticosteroids were tapered progressively, targeting the lowest effective dose or discontinuation by 6 months if inflammation remained controlled. The tapering schedule was as follows: for >40 mg/day, reduce by 10 mg every 1–2 weeks; for 20–40 mg/day, reduce by 5 mg every 1–2 weeks; for 10–20 mg/day, reduce by 2.5 mg every 1–2 weeks; and for <10 mg/day, reduce by 1–2.5 mg every 1–4 weeks. Adjustments were made based on clinical activity, with slower tapering or temporary dose increases allowed in cases of relapse.

Concomitant immunomodulators (e.g., methotrexate, cyclosporine A, mycophenolate mofetil) were continued as per the preexisting regimen, and no new agents were added during ADA therapy. Local intraocular steroids were not used unless required for severe relapses.

Before starting ADA, all patients underwent infection screening, including a mandatory interferon-γ release assay, with additional tuberculin skin testing in some, chest imaging, and serologic testing to exclude other active infections, per safety guidelines. Hepatitis B screening was performed using HBsAg, anti-HBs, and anti-HBc IgG [24].

### 4.4. Clinical Terminology

The following clinical parameters were used to assess disease activity and treatment response in this study. Intraocular inflammation was quantified using the Standardization of Uveitis Nomenclature (SUN) criteria [25]. AC cell grade and AC flare grade assess the degree of inflammatory cells and proteinaceous material in the anterior chamber, respectively, each graded from 0 (none) to 4+ (severe) under slit-lamp examination. Vitreous haze refers to the turbidity of the vitreous caused by inflammatory cells, also graded on a 0 to 4+ scale.

Anatomical outcomes were monitored by OCT. CMT represents retinal thickening due to edema, while SFCT, measured by enhanced depth imaging OCT, serves as an important biomarker of choroidal inflammation. BCVA was measured using a Snellen chart and converted to the logMAR scale for quantitative analysis.

### 4.5. Main Outcome Measures

**Figure 5:**
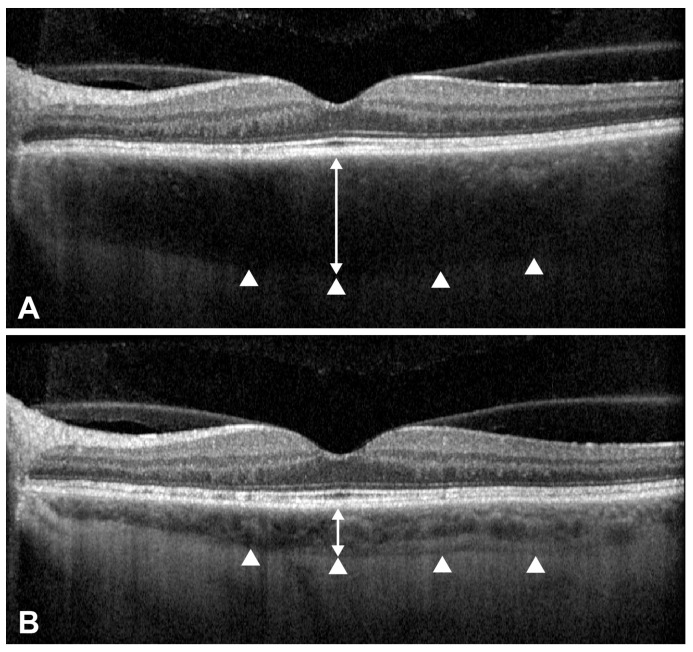
Enhanced depth imaging optical coherence tomography (EDI-OCT) findings of a representative eye with Vogt–Koyanagi–Harada (VKH) disease treated with adalimumab. (**A**) Baseline OCT image showing increased subfoveal choroidal thickness. (**B**) OCT image at 6 months after adalimumab treatment. Subfoveal choroidal thickness (double-headed arrow) was measured from the outer border of the hyperreflective retinal pigment epithelium (RPE) line to the chorioscleral interface (arrowheads) under the center of the fovea using the caliper function of the Heidelberg Eye Explorer software.

The primary outcome was the reduction in systemic corticosteroid dosage to evaluate the steroid-sparing effect of ADA. Daily prednisone-equivalent doses at baseline and each follow-up visit (up to 6 months) were recorded. Another key outcome was the change in the SFCT on OCT, serving as an anatomical marker of choroidal inflammation. SFCT was measured on enhanced depth imaging SD-OCT scans (Spectralis OCT, Heidelberg Engineering, Heidelberg, Germany) at baseline and 6 months after ADA initiation. It was defined as the perpendicular distance from the outer border of the retinal pigment epithelium to the chorioscleral interface under the fovea, measured manually using the caliper tool in the Heidelberg Eye Explorer software 1.10.2.0 (Figure 5).

Secondary outcomes included BCVA, AC cell grade, AC flare grade, vitreous haze grade, CMT, and frequency of inflammatory relapses. BCVA was assessed using a Snellen chart and converted to the logarithm of the minimal angle of resolution (logMAR) scale. For patients with severely reduced vision—such as “counting fingers,” “hand motion,” or “light perception”—corresponding numerical values were assigned for statistical analysis [26]. Intraocular inflammation was graded using standard uveitis criteria. AC cells were graded on a 0 to 4+ scale per the SUN criteria, with 0.5+ indicating 1–5 cells in a field size of 1 mm by 1 mm slit beam. AC flare was graded on a 0 to 4+ scale per SUN criteria (0 = none; 4+ = intense [fibrin or plastic aqueous]) [25]. CMT was defined as the central 1 mm retinal thickness on macular OCT. Uveitic relapses—defined as recurrences of active intraocular inflammation requiring escalation of therapy—were recorded for both the pre-ADA and ADA follow-up periods.

### 4.6. Statistical Analyses

All statistical analyses were performed using SPSS software version 29.0 (IBM Corp., Armonk, NY, USA). Continuous variables are presented as means ± standard deviations (SDs). The Shapiro–Wilk test was used to assess data normality and determine the appropriate method for paired comparisons. Paired *t*-tests were applied to normally distributed data, while Wilcoxon signed-rank tests were used for non-normally distributed or ordinal data (e.g., AC cell grade, flare grade, and annualized relapse rates). To ensure robustness of the small sample size (*N* = 8), both paired *t*-test and Wilcoxon test were used to analyze changes in the corticosteroid dose. The relationship between SFCT reduction and BCVA was assessed using Spearman rank correlation. Statistical significance was defined as *p* < 0.05. For analysis, BCVA values were converted to logMAR units.

Relapse was defined as new or worsening intraocular inflammation requiring treatment escalation (e.g., ≥2-step increase in AC cell grade, new chorioretinal lesions, or increased vitreous haze, per SUN criteria). To account for the variable follow-up durations among patients, ARR was calculated for both the pre-treatment and post-treatment periods. The formula used was: ARR = (Total number of relapses) ÷ (total observation months/12).

## 5. Conclusions

TNF-α inhibition with ADA—often used in combination with conventional immunomodulators—appears to be an effective and safe therapeutic option for managing inflammation, reducing corticosteroid burden, and decreasing relapse frequency in patients with chronic recurrent VKH disease refractory to conventional treatments. The reduction in SFCT may serve as a useful biomarker for monitoring treatment response. Although functional improvements in visual acuity were modest, achieving inflammation control while minimizing long-term corticosteroid exposure is critical for improving prognosis and quality of life in these patients. Further prospective multicenter studies are warranted to confirm these findings, optimize patient selection criteria, and establish the role of ADA in steroid-sparing or steroid-free regimens for this challenging patient group with chronic refractory VKH disease.

## Figures and Tables

**Table 1:** Baseline Characteristics of Patients with Refractory VKH Disease (*N* = 8 Patients).

Characteristics	Value
Demographics	
Age, mean ± SD (range), years	47.6 ± 9.5 (30–59)
Sex, male:female	3:5
Follow-up duration, months	38.75 ± 50.01 (6–84)
Baseline treatment	
Systemic Corticosteroids, number of patients (%)	6 (75%)
Mean dose, mg/day	14.7 ± 14.0
Concurrent immunomodulator, number of patients	7 (87.5%)
Baseline Ocular Data (N = 16 eyes)	
Baseline SFCT, µm	326.7 ± 129.1
Baseline BCVA, logMAR	0.36 ± 0.71
Previous annual relapse rate	3.61 ± 3.55
Steroid-intolerant eyes	2 (25%)

Values are presented as mean ± standard deviation (SD) or number (%). VKH, Vogt–Koyanagi–Harada; SFCT, subfoveal choroidal thickness; BCVA, best-corrected visual acuity; logMAR, logarithm of the minimum angle of resolution.

**Table 2:** Individual patient profiles: treatment history, adverse effects, and relapse rates before and after ADA in VKH disease.

Case	S/A	Medication at ADA Initiation	Adverse Effects of Steroid/IMT	Relapses Before ADA (*n*, Months, ARR)	Relapses After ADA (*n*, Months, ARR)
1	M/56	GC + MMF + CsA	Poor glycemic control, skin rash, arthralgia, CSC	1 (18 mo, 0.67/y)	0 (6 mo, 0/y)
2	M/44	GC + MMF + CsA	NA	3 (30 mo, 1.20/y)	0 (11 mo, 0/y)
3	M/55	GC + MMF	NA	3 (120 mo, 0.30/y)	0 (60 mo, 0/y)
4	F/48	GC + MMF + CsA	NA	3 (4 mo, 9.00/y)	1 (33 mo, 0.36/y)
5	F/59	None	Generalized edema, asthenia, mood changes, and psychosis	2 (3 mo, 8.00/y)	0 (18 mo, 0/y)
6	F/49	GC + MMF	NA	3 (6 mo, 6.00/y)	0 (84 mo, 0/y)
7	F/40	CsA	Cushing syndrome, alopecia	3 (12 mo, 3.00/y)	1 (39 mo, 0.31/y)
8	F/30	GC + MTX	NA	7 (117 mo, 0.72/y)	0 (6 mo, 0/y)

S/A, sex/age; IMT, immunomodulatory therapy; ADA, adalimumab; ARR, annualized relapse rate; GC, glucocorticoid; MMF, mycophenolate mofetil; CsA, cyclosporine A; MTX, methotrexate; NA, not applicable; CSC, central serous chorioretinopathy; mo, months; y, year. ARR was calculated as: (Total number of relapses) ÷ (total observation months/12). Follow-up durations varied among patients; ARR is used for a time-normalized comparison.

**Table 3:** Baseline and endpoint clinical measures in adalimumab-treated patients with recurrent Vogt–Koyanagi–Harada disease.

Clinical Parameters	Months		
	Baseline	At 6 Months	*p* Value
Primary outcome			
Prednisone dose (mg/day)	14.7± 14.0	4.1 ± 3.8	0.027
SFCT (µm)	326.7 ± 129.1	231.6 ± 72.9	<0.001
Correlation (SFCT × BCVA)			0.564 *
Secondary outcomes			
Anterior chamber cell	0.81 ± 0.66	0.09 ± 0.20	<0.001 *
Flare grade	0.38 ± 0.59	0.06 ± 0.17	0.059
BCVA (logMAR)	0.36 ± 0.71	0.32 ± 0.73	0.084
Central macular thickness	270.8 ± 54.2 µm	257.5 ± 29.9 µm	0.325

Data are presented as mean ± standard deviation. The prednisone dose was analyzed per patient (*N* = 8); all other clinical parameters were analyzed per eye (*N* = 16). * Spearman correlation between SFCT and BCVA changes at 6 months. SFCT, subfoveal choroidal thickness; BCVA, best-corrected visual acuity; logMAR, logarithm of the minimum angle of resolution.

## Data Availability

The complete dataset presented in this study is not publicly available due to privacy and ethical restrictions. However, data may be available from the corresponding author upon reasonable request. Anonymized key clinical parameters for all study participants are available in Supplementary Materials (Supplementary File S1).

## Supplementary Materials

The following supporting information can be downloaded at: https://www.mdpi.com/article/10.3390/ph18121848/s1, File S1: Anonymized key clinical parameters of study participants.

## Abbreviations

The following abbreviations are used in this manuscript:

AC	Anterior Chamber
ADA	Adalimumab
BCVA	Best-Corrected Visual Acuity
CMT	Central Macular Thickness
EDI	Enhanced depth imaging
IMT	Immunomodulatory therapy
logMAR	Logarithm of the Minimum Angle of Resolution
NIU	Noninfectious uveitis
OCT	Optical Coherence Tomography
SD-OCT	Spectral-Domain Optical Coherence Tomography
SFCT	Subfoveal Choroidal Thickness
SUN	Standardization of Uveitis Nomenclature
TNF-α	Tumor Necrosis Factor-alpha
VKH	Vogt–Koyanagi–Harada
